# Evaluation of the Best Method for Orthodontic Correction of Skeletal Deep Bites in Growing Patients: A Systematic Review

**DOI:** 10.7759/cureus.62666

**Published:** 2024-06-19

**Authors:** Omar Ahmad Rasol, Mohammad Y. Hajeer, Kinda Sultan, Mowaffak A. Ajaj, Ahmad S. Burhan, Samer T. Jaber, Ossama Aljabban

**Affiliations:** 1 Department of Orthodontics, Faculty of Dentistry, University of Damascus, Damascus, SYR; 2 Department of Orthodontics, Faculty of Dentistry, Al-Watanyia Private University, Hama, SYR; 3 Department of Endodontics and Restorative Dentistry, Faculty of Dentistry, University of Damascus, Damascus, SYR

**Keywords:** backward rotation of the mandible, eruption of posterior teeth, anterior bite plane, cover bite, closed bite, increasing the facial height, opening of the bite, horizontal growth pattern, skeletal deep bite, deep bite

## Abstract

This review aimed to evaluate the currently available evidence regarding the best method of correcting deep bites in growing patients. In September 2023, a search was conducted electronically across the following databases: PubMed®, Web of Science™, Scopus®, Embase®, Google™ Scholar, and Cochrane Library. In this systematic review, randomized control trials (RCTs), controlled clinical trials (CCTs), and cohort studies of growing patients with deep bite malocclusion who received treatment with the primary objective of treating the deep bite were included. Risk of bias of the included studies was assessed using two different tools; one tool was applied for RCTs and the other one for the CCTs and cohort studies. One RCT, one CCT, and one cohort study were included (85 patients). The flat fixed acrylic bite plane was superior in terms of duration of treatment when compared to the inclined fixed acrylic bite plane and the utility arch with posterior intermaxillary elastics. Limited evidence indicates that the inclined fixed acrylic bite plane causes a significant increase in the lower incisor inclination and a significant increase in the angle between the mandible and the anterior cranial base (SNB). However, limited evidence indicates that the utility arch with posterior intermaxillary elastics causes a significant decrease in the angle between the maxilla and the anterior cranial base (SNA). Regarding the vertical skeletal changes, it was found that the three methods were comparable; in each case, the vertical dimension of the face increased because of a significant increase in the lower first molar height. There is a need for further studies to strengthen the evidence of the treatment efficacy of the employed methods, with more RCTs to be conducted in this regard.

## Introduction and background

Deep bite is one of the most common vertical deformities that accompanies other types of malocclusions, with a prevalence of 13% in adults and 20% in growing patients in the U.S. population [1,2]. However, it is a condition of excessive overbite when the upper incisors' crowns cover more than a third of the lower ones in centric or habitual occlusion [3].

A deep bite is considered to be a clinical manifestation of dental or skeletal underlying problems [1]. Furthermore, the deep bite can cause serious effects at the level of the periodontal system, like gingival recession and looseness of teeth, tooth wear, and temporomandibular joint disorders, and can also affect the occlusion during anterior and lateral movement of the mandible [4]. However, skeletal deep bite occurs in short-faced patients with excessive forward rotation of the mandible and flat maxillary plane [5], and it usually accompanies class II division 2 malocclusion [6]. On the other hand, a cover bite is a complete deep bite usually associated with class II division 2 malocclusion. According to Walkow and Peck, in cover bite cases, the lower intercanine width was smaller than the control sample, which consisted of patients with class I, class II division 1 or division 2, or class III malocclusion, due to the assumption that the severe deep bite inhibits the anterior development of the mandibular dentoalveolar segment [7]. This statement has not been supported in other studies and is still controversial [8].

Dental deep bite occurs at the level of the teeth and alveolar processes due to over-eruption of the anterior teeth or premature loss of permanent teeth and lingual collapse of the maxillary or mandibular anterior teeth. According to Hotz and Mühlemann, deep bite can be divided into two categories: true deep bite and pseudo one [9]. True deep bite with a large freeway space, is caused by under-eruption of the molars, adequate freeway space will remain after extrusion of the molars. Whereas, pseudo deep bite with a small freeway space is caused by overeruption of the incisors where the molars have erupted fully. In such cases, it is not favorable to elevate the bite and extrude the molars. Because of significant posterior occlusion and muscular straining, any eruptive movement that extends beyond the interocclusal space may not be stable [1,9]. 

Deep bite malocclusions can be fixed in three basic ways: by extruding the posterior teeth, by intruding the incisors, or by tilting the incisors labially [1]. Extrusion of the molars is the most favorable approach for growing patients with a true or skeletal deep bite, provided that the interocclusal space remains unbroken [1]. Many appliances have been used for that end [1], and a fixed or removable anterior bite plane is one of these appliances. However, according to several studies, the anterior bite plane was considered an effective way to correct deep bite due to the extrusion of the posterior teeth and the relative intrusion of the lower incisors [10,11]. On the other hand, another study showed that the correction was due to the relative intrusion that occurred in the upper incisors and the extrusion of posterior teeth [12]. The most recent systematic review of anterior bite planes revealed that molar extrusion, particularly of the lower first permanent molars, was responsible for managing deep bite and that the anterior bite plane did not cause lower incisor intrusion [13]. Cervical headgear is another way to manage deep bite; it can be used alongside other appliances such as biteplates. A study showed that it was effective in reducing overbite and overjet and caused a mandibular backward rotation [14]. After reviewing the published literature, only a limited number of published systematic reviews on deep bite management were found. A systematic review published in 2018 about managing deep bite and retroclined upper front teeth showed that they did not identify any randomized controlled trial (RCT) or controlled clinical trial (CCT) that assessed the treatment of Class 2 div. II in children [15]. Another systematic review assessed the evidence of the effectiveness of the anterior bite planes in the correction of deep bite in growing patients. However, it only contained three CCTs, two of which were from theses that were not internationally published and lacked any RCTs [13]. Till now, no systematic review has been conducted to provide evidence regarding the most effective approach for managing deep bite in growing patients. Thus, the goal of this systematic review was to respond to the following specific review question: What is the best treatment modality to correct or alleviate skeletal deep bite in growing patients with different types of malocclusion?

## Review

Preliminary search and protocol registration

First, a PubMed pilot search was carried out before writing this systematic review's final protocol to ensure there were no similar ones and to identify any relevant articles. The Cochrane Handbook for Systematic Reviews of Interventions [16], the checklist, and the Preferred Reporting Items for Systematic Reviews and Meta-Analyses (PRISMA) [17,18] were used to write this systematic review.

Eligibility criteria

The present systematic review defined the exclusion and inclusion criteria for the included trials. For determining participants, interventions, comparisons, outcomes, and study design, the Population, Intervention, Comparison, Outcomes, and Study (PICOS) framework was utilized. The target population was growing patients in mixed dentition with a deep skeletal bite regardless of the malocclusion class. The intervention was any orthodontic treatment with the primary goal of managing the skeletal deep bite. In the case of comparative studies, patients in the comparison group should have received no orthodontic treatment at all or any appliance that differed from the one used in the intervention group. The primary outcomes of this criteria were the duration of deep bite correction, upper incisors vertical changes, upper incisors inclination, lower incisors vertical changes, lower incisors inclination, upper first molar vertical changes, lower first molar vertical changes, the angle between the mandible and the maxilla in the vertical plane, the angle between the anterior cranial base in the vertical plane, and the anterior facial height or the lower anterior facial height changes. The secondary outcomes were the angle between the maxilla and the anterior cranial base (SNA), the angle between the mandible and the anterior cranial base (SNB), and the angle between the mandible and maxilla in the horizontal plane (ANB). In this review, only RCTs, CCTs, and cohort studies were sought. No limitations concerning language or publication year were applied.

The excluded studies were the following: studies that did not differentiate between dental and skeletal deep bite, studies that did not have deep bite correction as their primary objective, studies that did not report a sample, studies with fewer than ten patients in the experimental group, editorials, case reports, case series reports, retrospective studies, personal opinions, reviews, and technique description articles.

Sources of information

The search strategy's keywords are listed in Appendix 1. The primary search was carried out without a time constraint in September 2023 by two reviewers (OAR and MYH) using PubMed®, Web of Science™, Scopus®, Embase®, Google™Scholar, and Cochrane Library. A manual search of the bibliographies of all the included articles was conducted to find more relevant papers.

Search strategy and study selection

There were two stages involved in the process of selecting articles. The first step involved the two reviewers (OAR and MYH) independently looking over the abstracts and titles of the articles found via the electronic search. In the second step of the review process, the full texts of the eligible articles were evaluated by the same two reviewers. The review did not include any articles that did not meet the inclusion criteria. The reviewers resolved conflicts through discussion and reached out to the third author (KS) until an agreement was reached.

Data collection process

Two reviewers (OAR and MYH) took data from the included trials and organized them into tables. The following data were included: general information (authors' names, publication year), study design, number of patients, mean age, malocclusion type, intervention type, follow-up period, treatment duration, and outcomes. In cases of disagreement, the two reviewers talked it over and collaborated with the third author (KS) until they reached a consensus.

Evaluation of the risk of bias in specific studies

Two reviewers (OAR and MYH) independently evaluated the risk of bias for each included study using the Cochrane tool for risk of bias (ROB2) for the RCTs [19] and the Risk of Bias In Non-randomized Studies of Interventions (ROBINS-I) tool for the CCTs [20]. The two reviewers' assessments were then compared; if there were differences, the reviewers worked with the third review author (KS) to resolve them until they could agree on a conclusion. The risk of bias in the following domains was rated as "low," "high," or "some concerns" for randomized trials: bias resulting from the randomization process, bias resulting from deviations from the planned interventions, bias brought by missing outcome data, bias in the measurement of outcome, and bias in the selection of the result that was reported. The selected studies were evaluated for overall risk of bias in the following manner: "low risk of bias" if all fields were evaluated as "at low risk of bias"; "some concerns" if one or more domains were deemed to have "some concern" but not at "at high risk of bias"; "high risk of bias" if at least one or more fields were evaluated as "at high risk of bias" or there were some concerns for multiple domains In a way that significantly reduces confidence in the outcome. However, for non-randomized trials, the following domains were assessed: confounding-related bias, bias in the way study participants were chosen, bias in how interventions were categorized, bias due to deviations from planned interventions, bias caused by missing data, bias in the way outcomes were measured, and bias in the way the reported result was selected. The total risk-of-bias evaluation of the chosen studies was assessed in the way that follows: "low risk of bias" if every domain was assessed as "at low risk of bias"; "moderate risk of bias" if every domain was assessed as " low or moderate risk of bias"; "serious risk of bias" if at least one domain was evaluated as "serious risk of bias" but not at critical risk of bias in any domain; "critical risk of bias" if at least one domain was evaluated as "critical risk of bias"; "no information" if there was no obvious indication that the study was "at serious or critical risk of bias" and there was a paucity of information in one or more key domains of bias.

Results

Study Selection and the Literature Review

**Figure 1:**
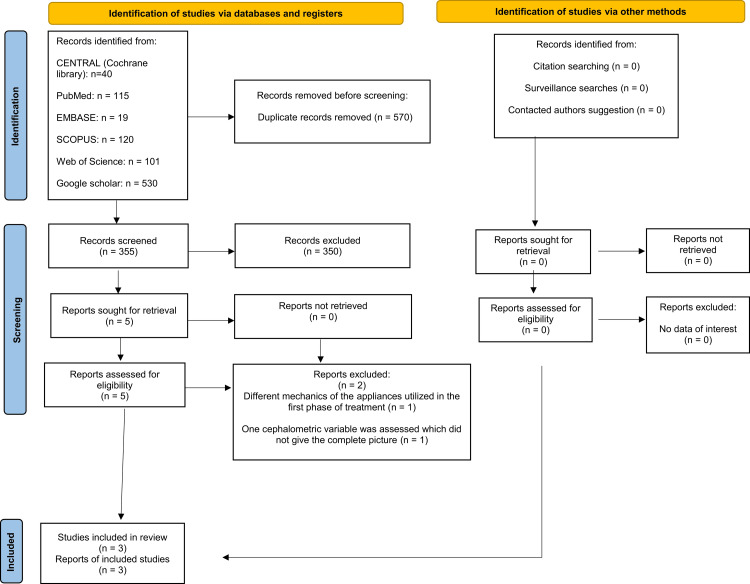
The Preferred Reporting Items for Systematic Reviews and Meta-Analyses (PRISMA) flow diagram of the reviewing process.

Nine hundred twenty-five articles were found following the computerized search. The number was reduced to 355 after removing the duplicates. After reading the titles and abstracts of the remaining papers, those that did not meet the eligibility criteria were excluded; therefore, five potentially relevant articles remained. After reading the full text of these five articles, two studies did not match the inclusion criteria. Therefore, three studies (one randomized controlled trial, one controlled clinical trial, and one cohort study) were included in this systematic review. The PRISMA flow diagram is given in Figure 1.

Characteristics of the Included Studies 

The characteristics of the three trials included in this systematic review are listed in Table 1. One randomized controlled trial [12], one controlled clinical trial [10], and one cohort study [11] were included in this systematic review. The total number of patients in each study was 85, with a mean age range from 9.9 to 11.3 years. All studies included both genders (47 males and 38 females). There were more males than females in two studies [10,12]. However, In one study, there were more females than males [11].

**Table 1:** Characteristics of included studies in this systematic review C: control group, T: treated group, m: male, f: female, CCT: controlled clinical trial, RCT: randomized controlled trials, MM: the angle between the mandible and the maxilla in the vertical plane, LFH: lower anterior facial height changes, AFH: the anterior facial height,  SNA: the angle between the maxilla and the anterior cranial base, SNB: the angle between the mandible and the anterior cranial base, ANB: the angle between the mandible and maxilla in the horizontal plane, F: fixed anterior acrylic bite plane group, U[Ma1]: utility arch group.

Authors	Study design	Number of patients	Mean age	Malocclusion type/inclusion criteria	Intervention	Outcomes	Effective treatment stoppage standard
Forsberg and Hellsing, 1984 [10]	CCT	40 patients: C, 20 patients (12 m, 8 f); T, 20 (12 m, 8 f)	C, 11.3±1.5 years; T, 11.3±1.4 years	Patients with deep overbite, the lower incisors occluding with the palatal mucosa.	Intervention group: lingual arch with an anterior acrylic bite plane which was fixed to the molar bands. Control group: Untreated	Primary outcomes: the duration of deep bite correction, upper and lower incisors vertical changes, upper and lower incisors inclination, upper and lower first molars vertical changes, MM angle, and LFH or AFH changes. Secondary outcomes: SNA, SNB, ANB	When the first molars gained contact so that an articulating foil with a thickness of 8 microns between these teeth in centric occlusion could not be removed.
Akarsu-Guven et al., 2010 [11]	Cohort	17 patients (8 m, 9 f)	9.9 ± 0.9 years	Class II malocclusion, deep bite more than 3 mm, brachyfacial growth pattern.	Fixed inclined acrylic bite plane	Primary outcomes: the duration of deep bite correction, upper and lower incisors vertical changes, upper and lower incisors inclination, upper and lower first molars vertical changes, MM angle, and LFH or AFH changes. Secondary outcomes: SNA, SNB, ANB	When the bite was opened and the class I molar relationship was achieved.
Alsawaf and Rajah, 2023 [12]	RCT	28 patients: F, 14 (7 m, 7 f); U, 14 (8 m, 6 f)	F: 10.67±1.25 years; U: 10.65±0.97 years	Skeletal deep bite, Skeletal Class I or mild to moderate Class II, Retroclined upper incisors, Overbite more than 40%.	Control group: fixed anterior acrylic bite plane. U group: Utility arch with vertical posterior inter-maxillary elastics.	Primary outcomes: the duration of deep bite correction, upper and lower incisors vertical changes, upper and lower incisors inclination, upper and lower first molars vertical changes, MM angle, and LFH or AFH changes. Secondary outcomes: SNA, SNB, ANB	When the overbite reached a normal value (40%)

One study investigated the flat fixed acrylic anterior bite plane [10], while another investigated the inclined fixed acrylic anterior bite plane [11]. However, the third one compared the fixed anterior acrylic bite plane and the utility arch with posterior inter-maxillary elastics [12]. The three trials used lateral cephalograms and clinical examinations as diagnostic tools to identify potential patients [10-12]. However, plaster casts were used in one study to measure the distance between the upper and lower first molars on the patient's right side at each visit [10].

One study included patients with deep overbite where the lower incisors occluded with the palatal mucosa, class II division 1 or class II division 2 malocclusion, with a mean age of 11.3 years. It was unclear whether the deep bite was skeletal or dental in this study [10], while another study included patients with class II malocclusion and skeletal deep bite more than 3 mm with a mean age of 9.9 years [11]. However, the third one included patients with a skeletal deep bite of more than 40% and skeletal class I or mild class II malocclusion with a mean age of 10.66 years [12].

The effective treatment cessation differed between studies. According to one study, the first molars in centric occlusion ended the effective treatment period, making it impossible to remove an articulating foil between them that was 8 microns thick without being torn [10]. However, according to a different study, the therapy reached its endpoint when the bite had opened, and the class I molar relationship was established [11]. The third study, however, concluded that the cessation of treatment should occur when the overbite reaches the normal value (40%) [12].

The variables were similar in the three studies. Upper and lower incisors vertical changes, upper and lower incisors inclination, upper and lower first molars vertical changes, the SNA, the SNB, the angle between the maxilla and the mandible in the vertical plane (MM) and the ANB, and lower anterior facial height were all examined in all three trials [10-12].

Risk of Bias of Included Trials

**Figure 2:**
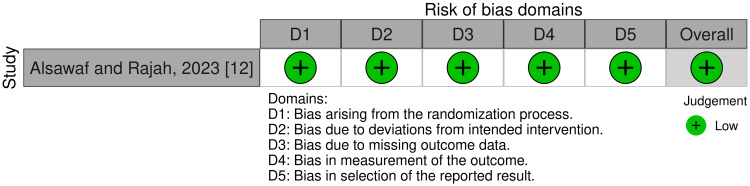
Risk of bias of the included randomized controlled trial in this review.

**Figure 3:**
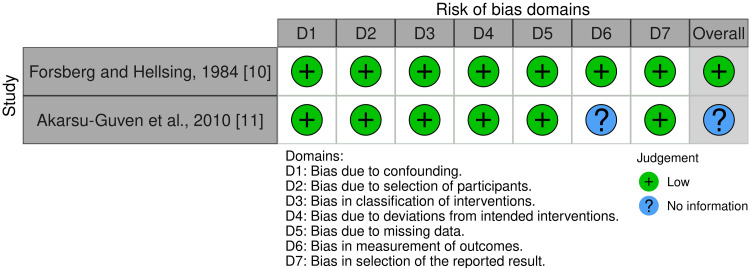
Risk of bias of the included non-randomized clinical trials.

One randomized trial was classified as low risk of bias [12]. Of the non-randomized trials, one study was classified as low risk of bias [10], and the other one was also classified as "no information" due to the lack of information about the outcomes assessors [11]. The total risk of bias of the included studies is summarized in Figure 2 and Figure 3, while Appendices 2 and 3 provide the rationale for each decision.

Effects of Interventions

Table 2 summarizes the collected findings of the retrieved studies.

**Table 2:** The summarized outcomes of the included studies * All the values mentioned in this table are in the form of mean values ± standard deviations. T: treated group, C: control group, S: significant, NS: nonsignificant, UIP: upper incisor inclination, VCU1: vertical change in the incisal edge of the upper incisor, LIP: Lower incisor inclination, VCL1: vertical change in the incisal edge of the lower incisor, VCU6: vertical change in the upper first molar, VCL6: vertical change in the lower first molar, SNA: the angle between the anterior cranial base and the maxilla, SNB: the angle between the anterior cranial base and the mandible. ANB: the angle between the maxilla and the mandible in the sagittal plane, MM: the angle between the maxillary plane and the mandibular plane, LAFH: lower anterior facial height, U: utility arch, F: fixed anterior acrylic bite plane.

Authors	Effective phase duration*	Dental changes						Skeletal changes				
		Anterior teeth changes				Posterior teeth changes		Sagittal			Vertical	
		UIP	VCU1	LIP	VCL1	VCU6	VCL6	SNA	SNB	ANB	MM	LAFH
Forsberg and Hellsing, 1984 [10]	3.6 ± 1.0 months	T: 1.8±1.9; C: 1.1±2.4^NS^	T: 0.1±0.6; C: 0.3±0.5^NS^	T: 0.9±0.9; C: 0.3±1.4^NS^	T: 0.1±0.5; C: 0.9±0.7^S^	T: 1.3±0.6; C: 1.0±0.5^NS ^	T: 1.4±0.7; C: 0.7±0.6^S^	T: 0.3±0.6; C: 0.0±0.7^NS^	T: 0.3±0.7; C:-0.2±0.7^NS^	T: 0.3±0.7; C: 0.2±0.7^NS^	T: 1.0±1.0; C: -0.5±0.9^S ^	T: 2.9±0.9; C: 1.2±1.2^S^
Akarsu-Guven et al., 2010 [11]	8.5 ± 2.1 months	4.8±8.05^S^	0.0±1.9^NS^	5.3± 5.62^S^	-0.3±2.29^NS^	0.3±2.59^NS^	2.3±2.81^S^	0.4±3.02^NS^	1.2±2.91^S^	-0.7±1.50^S^	1.6± 4.35^S^	4.3± 3.64^S^
Alsawaf and Rajah, 2023 [12]	F: 7.22 ± 2.63 months; U: 8.16 ± 2.42 months	U: 6.6±4.32; F: 5.9±5.42^NS ^	U: 0.28±0.89; F: 1.45±1.80^S^	U: 3.39±3.05; F: 2.0±5.1^NS^	U: 0.38±0.83; F: 0.3±1.56^NS^	U: 0.51± 1.22; F: 0.2± 1.46^NS^	U: 0.6±1.36; F: 0.5± 1.12^NS^	U: 0.78±1.24; F: 0.3±1.56^S^	U: 0.20±0.49; F: 0.22± 1.5^NS^	U: -0.58± 0.93; F: 0.09± 0.92^NS^	U: 1.5±1.8; F: 2.81±1.74^NS^	U: 1.8±2.9; F: 2.87±3.54^NS^

Primary outcomes: The duration of deep bite correction using a flat fixed acrylic bite plane was found to be 3.6±1.0 months in Forsberg and Hellsing's study [10], whereas Alsaswaf and Rajah compared it to the utility arch, which took a mean of 8.16±2.42 months while the flat fixed bite plane took a mean of 7.22±2.63 months [12]. In the third study, the duration was 8.5±2.1 months using an inclined fixed acrylic bite plane [11].

Dental changes: Forsberg and Hellsing investigated the effect of the flat fixed acrylic bite plane compared to an untreated group. The two groups had a statistically significant difference regarding the lower incisor height. They found no significantly different change in the treated group, while it increased in the control group. The lower first molar showed a higher amount of eruption compared to the control group [10]. Akarsu-Guven et al. investigated the effect of the inclined fixed acrylic bite plane. They found a statistically significant increase in the lower incisor inclination and first molar height [11]. Alsaswaf and Rajah compared the flat fixed acrylic bite plane and the utility arch with posterior inter-maxillary elastic. Both groups showed a statistically significant increase in the upper incisor inclination, with no statistically significant difference between groups. In the utility arch group, there was a statistically significant increase in the lower incisor inclination with no statistically significant difference between groups. In the bite plane group, the upper incisor height decreased significantly compared to the other group [12].

Skeletal changes: In two studies, there was a statistically significant increase in the angle between the mandible and the anterior cranial base [10,11], while in one study, there was a statistically significant difference in each group and an insignificant difference between the two groups [12]. The angle between the mandible and the maxilla in the vertical plane was assessed in two studies. In one study, the angle increased significantly in the treated group [10], while in the other one, the angle increased in both groups with no significant difference between groups [12]. The lower anterior facial height was assessed in two studies, and it increased significantly in both [10,11], while the third study assessed the anterior facial height, which increased significantly in both groups with no significant difference between groups [12].

Secondary outcomes: Forsberg and Hellsing reported that there were insignificant differences in the maxillary sagittal positioning angle, the mandibular sagittal positioning angle, and the skeletal sagittal relationship angle [10], whereas Akarsu-Guven et al. reported that there was a statistically significant increase in the mandibular sagittal positioning angle and a decrease in the skeletal sagittal relationship angle with an insignificant change in the maxillary sagittal positioning angle [11]. However, in Alsaswaf and Rajah's study, there was an insignificant change in the three angles in the bite plane group, while the maxillary sagittal positioning angle and the skeletal sagittal relationship angle decreased significantly in the utility arch group, with an insignificant change in the mandibular sagittal positioning angle. The decrease in the maxillary sagittal positioning angle between the two groups was statistically significant [12].

Discussion

When reviewing the current literature, one notices a significant lack of studies on deep bite malocclusion in growing patients, leading to a lack of systematic reviews and scientific evidence. According to this systematic review, whether flat or inclined, the anterior acrylic bite plane seems to be the dominant appliance for deep bite correction in growing patients. Forsberg and Hellsing found that the flat anterior acrylic fixed bite plane was effective in the management of deep bites when compared to the untreated group [10]. Akarsu-Guven et al. used the inclined anterior acrylic bite plane, effectively treating deep bite and class II malocclusion [11]. However, Alsawaf and Rajah also used the flat fixed anterior bite plane. They compared it with the utility arch and posterior intermaxillary elastics. Both appliances were effective in growing deep bite patients, where the flat fixed anterior bite plane outperformed the utility arch regarding treatment duration [12].

According to Forsberg and Hellsing, the lower incisor height did not significantly increase in the treated group, while it significantly increased in the untreated group [10]. Akarsu-Guven et al. reported that there was an insignificant difference [11]. Alsawaf and Rajah found no significant change in both groups [12]. This may be explained by the fact that in the Forsberg and Hellsing study, the bite plate inhibited the natural eruption of the lower incisors, but the other group included untreated patients [10]. In addition, this study did not report if the deep bite was of skeletal origin, which may have affected the duration of deep bite correction, taking a noticeably shorter time than the other studies. The lower first molar showed significant eruption in the three studies. Alsawaf and Rajah’s study showed significant eruption in each group, with insignificant differences between groups [12]. This might be explained by the posterior occlusal clearance that the anterior bite plane generated and enabled the molars to erupt freely, as well as the elastomeric forces that compelled the molars to erupt in the utility arch group in Alsawaf and Rajah's study.

The three studies showed a significant increase in the vertical parameters [10-12]. This may indicate that the current methods will lead to the eruption of the lower first molars, which will alter the short face type by increasing the vertical dimension.

In terms of sagittal parameters, Forsberg and Hellsing did not find any significant changes [10]. Akarsu-Guven et al. reported that the mandibular sagittal positioning angle increased significantly [11]. This may be explained by using an inclined anterior bite plane that keeps the mandible forward and serves as a functional appliance for class II malocclusion and its use in managing deep bite malocclusion. Alsawaf and Rajah reported that the maxillary sagittal positioning angle decreased significantly in the utility arch group. This finding might be explained by the upper incisors' leveling and alignment before applying the utility arch, which causes the roots to move lingually and the A point to travel backward [12].

Limitations of the Current Systematic Review

As mentioned before, the main limitation of this systematic review is the lack of studies dealing with managing skeletal deep bite malocclusion in growing patients. Only one RCT was included; the other studies were one cohort study and one CCT. This reduced confidence in the results and precluded doing a meta-analysis.

## Conclusions

Given the limited number of research comparing the various approaches, the evidence and data about the superiority of one deep bite management approach over another in growing patients is still insufficient. Regarding treatment duration, limited evidence indicates that the flat fixed anterior bite plane requires less time than the other two techniques. Regarding dental changes, limited evidence suggests that the inclined fixed anterior bite plane causes a significant increase in the lower incisor inclination. The three approaches have no clinically important differences regarding the vertical skeletal changes. Nevertheless, regarding sagittal vertical changes, limited evidence indicates that the inclined fixed anterior bite plane causes a significant increase in the mandibular sagittal positioning angle, while the utility arch with posterior intermaxillary elastics causes a significant decrease in the maxillary sagittal positioning angle. Therefore, more well-planned RCTs with good randomization and patient selection processes and innovative methods and appliances for this specific malocclusion are required in the future.

## Appendices

Appendix 1 

**Table 3:** The electronic search strategy used in the current systematic review

PubMed®	#1 (deep bite OR deep overbite OR increased overbite OR deep-bite OR excessive overbite OR decreased vertical dimension) #2 (deep bite management OR deep bite correction OR deep bite treatment) #3 (bite plane OR anterior bite plane OR fixed bite plane OR removable bite plane OR bite turbo OR flat bite plane OR inclined bite plane OR functional appliance OR utility arch) #4 (growing patients OR mixed dentition OR children) #5 #1 AND #2 AND #3 AND #4
CENTRAL (The Cochrane Library)	#1 (deep bite OR deep overbite OR increased overbite OR deep-bite OR excessive overbite OR decreased vertical dimension) #2 (deep bite management OR deep bite correction OR deep bite treatment) #3 (bite plane OR anterior bite plane OR fixed bite plane OR removable bite plane OR bite turbo OR flat bite plane OR inclined bite plane OR functional appliance OR utility arch) #4 (growing patients OR mixed dentition OR children) #5 #1 AND #2 AND #3 AND #4
Web of Science™	#1TS= (deep bite OR deep overbite OR increased overbite OR deep-bite OR excessive overbite OR decreased vertical dimension) #2TS= (deep bite management OR deep bite correction OR deep bite treatment) #3TS= (bite plane OR anterior bite plane OR fixed bite plane OR removable bite plane OR bite turbo OR flat bite plane OR inclined bite plane OR functional appliance OR utility arch) #4TS= (growing patients OR mixed dentition OR children) #5 #1 AND #2 AND #3 AND #4
Scopus®	#1 TITLE ABS-KEY (deep bite OR deep overbite OR increased overbite OR deep-bite OR excessive overbite OR decreased vertical dimension) #2 TITLE ABS-KEY (deep bite management OR deep bite correction OR deep bite treatment) #3 TITLE ABS-KEY (bite plane OR anterior bite plane OR fixed bite plane OR removable bite plane OR bite turbo OR flat bite plane OR inclined bite plane OR functional appliance OR utility arch) #4 TITLE ABS-KEY (growing patients OR mixed dentition OR children) #5 #1 AND #2 AND #3 AND #4
EMBASE®	#1 (deep bite OR deep overbite OR increased overbite OR deep-bite OR excessive overbite OR decreased vertical dimension) #2 (deep bite management OR deep bite correction OR deep bite treatment) #3 (bite plane OR anterior bite plane OR fixed bite plane OR removable bite plane OR bite turbo OR flat bite plane OR inclined bite plane OR functional appliance OR utility arch) #4 (growing patients OR mixed dentition OR children) #5 #1 AND #2 AND #3 AND #4
Google™ scholar	(deep bite OR deep overbite OR increased overbite OR deep-bite OR excessive overbite OR decreased vertical dimension) AND (deep bite management OR deep bite correction OR deep bite treatment) AND (bite plane OR anterior bite plane OR fixed bite plane OR removable bite plane OR bite turbo OR flat bite plane OR inclined bite plane OR functional appliance OR utility arch) AND (growing patients OR mixed dentition OR children)

 Appendix 2

**Table 4:** The risk of bias in the included randomized controlled trial.

Study	Randomization process	Deviations from intended interventions	Missing outcome data	Measurement of the outcome	Selection of the reported result	Over-all bias
Alsawaf and Rajah, 2023 [12]	Low: The randomization sequence was computer-generated. The allocation sequence was concealed using sequentially numbered, opaque, sealed envelopes.	Low: We did not detect deviations from the intended intervention arising from the trial context.	Low: The number of patients completing the trial was consistent with the sample size needed for the study.	Low: The outcome assessor was blinded.	Low: The study was registered with the German Clinical Trials Register (https://www.drks.de/drks_web/navigate.do?navigationId¼trial.html&TRIAL_ID¼DRKS00028870), and the outcomes mentioned in the protocol have been reported.	LOW

Appendix 3

**Table 5:** The risk of bias of the included non-randomized studies.

Study	Bias due to confounding	Bias in the selection of participants in the study	Bias in the classification of interventions	Bias due to deviations from intended interventions	Bias due to missing data	Bias in the measurement of outcomes	Bias in the selection of the reported result	Overall bias
Forsberg and Hellsing, 1984 [10]	Low: There were no obvious confounding factors that indicated a clear risk of bias.	Low: The treatment was performed on 20 randomly selected patients, and this group was compared with a control group comprising 20 untreated patients matching the intervention group in terms of age, sex, and type of malocclusion.	Low: There was an intervention group and a well-matched control group of untreated patients.	Low: There was only an intervention group, and no deviation from the intended intervention was spotted.	Low: The data were reasonably complete.	Low: The measurements were made with a digital electronic measuring device to a precision of 0.1 mm or 0.1 degree.	Low: The study is comparable to a well-performed randomized trial in this domain.	LOW
Akarsu-Guven et al., 2010 [11]	Low: There were no obvious confounding factors that indicated a clear risk of bias.	Low: The start of the intervention and the follow-up were coordinated with the participants, who were chosen before the intervention began.	Low: The intervention group was clearly defined.	Low: Any deviations from the intended intervention reflected usual practice.	Low: The data were reasonably complete.	NI: The outcome assessor's identity and awareness of the intervention were unclear.	Low: The ethics committee decision dated June 23, 2004, with the registration number LUT 04/30 was received from the Ethics Committee of Medical, Surgical, and Drug Research at Hacettepe University, and the outcomes mentioned in the protocol have been reported.	NI
ni NI: no information								
